# Transient Subclinical Hypothyroidism and Acute Suicidal Ideation Following Treatment with Escitalopram

**DOI:** 10.7759/cureus.5144

**Published:** 2019-07-15

**Authors:** Izza Bazigh, Sanjaya Dharmapuri, Rosario M Cosme

**Affiliations:** 1 Psychiatry, Rush University Medical Center, Chicago, USA; 2 Child Psychiatry, Rush University Medical Center, Chicago, USA

**Keywords:** selective serotonin reuptake inhibitors (ssri), subclinical hypothyroidism, ecitalopram, thyroid stimulating hormone (tsh), suicidal ideation, adolescent, side effects

## Abstract

Thyroid function abnormalities after the use of selective serotonin reuptake inhibitors (SSRIs) have been reported in the extant literature, but the strength of this correlation is unclear and a commentary on its clinical significance is necessary.

The one-week hospital course of a 16-year-old male presenting with worsening of major depressive disorder after the initiation of escitalopram was significant for the development of transient subclinical hypothyroidism (SCH). An analysis of the aberrant thyroid indices in the setting of escitalopram use was pursued. Data from previous studies suggesting similar events were reviewed in order to better characterize the nature of this association.

The exact diagnostic criterion for SCH in the pediatric population has been undergoing reform and, based on the newer suggestions, our patient was assessed to have transient SCH. Thyroid hormone derangement following SSRI use has been reported in the past; however, the clinical implications of developing transient SCH in adolescent patients with major depression who are taking SSRIs is still unclear and not well understood at this time.

The importance of having a better understanding of this potential interaction is marked by both the increased risk for suicidal ideation in the pediatric population with the use of SSRIs, as well as the confounding overlap of symptom presentation between hypothyroidism and major depressive disorder.

## Introduction

The clinical implications of developing transient subclinical hypothyroidism (SCH) in patients with major depression who are taking selective serotonin reuptake inhibitors (SSRIs) have received little attention. The importance of having a better understanding of this potential interaction is marked by both the increased risk for suicidal ideation in the pediatric population with the use of SSRIs, as well as the confounding overlap of symptom presentation between hypothyroidism and major depressive disorder. SSRIs carry a Federal Drug Administration (FDA) black box warning for the increased risk of suicidal ideation in the pediatric population; yet, there is no current evidence-based understanding as to why this occurs.

## Case presentation

DR was a 16-year-old adopted male who presented to the emergency department with the acute onset of suicidal ideation, culminating into a suicide attempt in the setting of recent worsening of depression following the initiation of escitalopram, 10 mg per day, 10 days earlier. He had no known medical history but did have a previous psychiatric history of attention deficit hyperactive disorder (ADHD) and major depressive episode (MDE). The patient was given a presumptive diagnosis of major depressive disorder (MDD), single and severe episode without psychotic features, and admitted for psychiatric stabilization.

DR’s depressive symptoms began three months prior to his admission. In reference to that, he reported low mood, poor self-esteem manifesting in self-derogatory thoughts, guilt, anhedonia, social isolation/withdrawal, sleep disturbances, fatigue, poor concentration, academic performance decline, decreased appetite, and passive suicidal ideation. His depressive symptoms worsened 10 days before admission, noticeably in the context of the initiation of escitalopram that was prescribed by his primary care provider. 

The adoptive parents confirmed DR’s history, providing supporting collateral information which reiterated their impression of DR as being depressed but stable prior to initiation of escitalopram. They reported that his symptoms were similar to a previous transient MDE when he was transitioning from middle school to high school. During that episode, DR pursued outpatient psychotherapy and the quick resolution of symptoms led to a reformulation of diagnosis as an adjustment reaction. The adoptive parents had little information on DR’s biologic family medical history aside from noting that his biologic mother died of hypothermia, as well as that DR was born in Siberia, Russia and adopted at the age of three.

During his current presentation, his affect was flat, with a restricted range and low intensity. He expressed negativistic thinking and described feeling like “a burden on everyone,” as well as “not valuable or smart.” There were no clinical signs or symptoms of hypomania, mania, or psychosis and he denied ever having homicidal ideation or auditory/visual hallucinations. He denied any previous or active history of tobacco, alcohol, or illicit substance use. DR did acknowledge having multiple psychosocial stressors, including the recent termination of a romantic relationship. However, he did not attribute the acute onset of suicidal ideation to any particular triggering event other than a notable increase in rumination over negativistic thinking after starting escitalopram. 

DR’s workup after admission included baseline complete blood count (CBC), comprehensive metabolic panel (CMP), thyroid-stimulating hormone (TSH), vitamin D level, electrocardiography (ECG), and medication evaluation. The escitalopram was tapered towards discontinuation, given the concerns for the possibility of drug-induced suicidal ideation, and a trial of fluoxetine was then initiated. His lab work from the time of admission was remarkable only for a mild elevation of the TSH at 3.334 uIU/mL and a mild elevation of the low-density lipoprotein (LDL) at 103 mg/dL. Upon gathering further information during initial interviews after admission, it was revealed that DR had concerns about recently developing constipation, cold intolerance, and observing that his hair appeared to be falling out easily overnight. Based on these reports, as well as his laboratory results showing mild aberrations in thyroid function, an additional workup for consideration of hypothyroidism was pursued, including repeat TSH, free T3/T4, anti-thyroperoxidase (anti-TPO) antibody, anti-thyroglobulin (anti-TG) antibody, and lipid panel. His repeat thyroid labs (taken within three days of admission) showed that T4 was in the lower end of normal limits/borderline deficiency, T3 was in the upper end of normal limits, and TSH was trending down within normal limits, while anti-TPO and anti-TG antibodies were not present (See Table 1). The lipid panel revealed normal total cholesterol, triglyceride, and high-density lipoprotein (HDL) level, but a mildly elevated LDL level. DR’s subjective reports of symptoms were concerning, but the results of the lab work, although aberrant, were not revealing of a primary thyroid function abnormality and a consult with endocrinology was not pursued during his admission and rather given as an outpatient referral if his symptoms persisted. 

**Table 1:** Thyroid Laboratory Values (* Latest recommendations suggest TSH upper limit to be 2.5 uIU/ml) T3: triiodothyronine; TSH: thyroid-stimulating hormone

Reference Range and Units	(Tg)Ab (O.O - 4.1 IU/L)	Tg (ng/mL)	Free thyroxine (0.7 - 1.5 ng/dL)	T3, Free (1.7 - 3.7 pg/mL)	TSH (0.350 - 4.940 uIU/mL)*
On admission					3.344
On 3rd day of admission	< 3.0	2.5	0.9	2.9	2.556

Within five days of transitioning to fluoxetine and the discontinuation of escitalopram, DR began showing marked improvement in depressive symptoms. He reported his mood was much improved, he had better appetite, less fatigue, improved sleep, and his suicidal ideation remitted. He noted a specific improvement in negativistic thinking and the absence of feelings of guilt which had previously been ruminative. Per staff reports, he was observed to have a progressive improvement in motivation and social participation within the milieu and during therapeutic programs. Upon acute stabilization, follow-up care was established outside of the hospital system, and he was discharged within seven days of his admission.

## Discussion

Thyroid function abnormalities after the use of SSRIs have been reported in the extant literature but the clinical implications are still unclear. Normal TSH range is commonly accepted to be within 0.35 mU/l and 4.9 mU/l and experts suggest that the upper limit should be adjusted for the pediatric population to 2.5 mU/L [1-2]. DR had no known thyroid function abnormalities or other medical illness prior to the initiation of an SSRI. Based on the results of his thyroid function panel, and given the absence of anti-TPO/anti-TG antibodies, DR did not meet criteria for a diagnosis of primary hypothyroidism. While DR had very mild aberrations in his thyroid function panel results, he still met criteria for a diagnosis of SCH at the time of his admission based upon the elevation of his TSH, which appears to have been transient and resolved by the time of his discharge. SCH is defined by an elevation in TSH despite normal levels of serum-free thyroxine, without overt clinical symptoms [3]. SCH may be a result of medical illness or a transient reaction to a homeostatic process and has been associated with a variety of affective syndromes. There is also evidence in the literature to suggest a potential correlation between the initiation of SSRIs and the development of thyroid function abnormalities, such as was seen in DR, and appears to best explain the transience and resolution of his SCH after discontinuation of the SSRI [4-5].

It is commonly accepted that SSRI use in adolescents is marked by an initial increase in the risk of suicidal ideation and attempts possibly due to a transient increase in agitation or activation, which may induce already depressed individuals to follow through on existing suicidal thoughts or plans [6-7]. While there are studies that attempt to document this observation, there is a paucity of studies undertaken to uncover the neurobiological basis underlying this drug response in adolescents. Other alternative hypotheses assert a largely unfalsifiable logic that there is a direct SSRI cause-effect to negativistic thinking in some individuals. However, a specific pathophysiological mechanism(s) that underlie multiple systemic effects, which occurs as a result of the initiation of SSRIs, has yet to be fully characterized.

## Conclusions

Our case report aims to implant the idea of a possible cause-effect link between two macroscopic events observed after SSRI intake in children, i.e, the endocrine change and the neuropsychiatric change, and calls for an exploration of a possible cross-talk on the cellular level that may add evidence to this cause-effect relationship. Either way, a clinical commentary on the endocrine derangement following initiation of SSRIs and its potential role in enhancing the risk for worsening of depression in adolescents is warranted.
